# The copy number variation and stroke (CaNVAS) risk and outcome study

**DOI:** 10.1371/journal.pone.0248791

**Published:** 2021-04-19

**Authors:** John W. Cole, Taiwo Adigun, Rufus Akinyemi, Onoja Matthew Akpa, Steven Bell, Bowang Chen, Jordi Jimenez Conde, Uxue Lazcano Dobao, Israel Fernandez, Myriam Fornage, Cristina Gallego-Fabrega, Christina Jern, Michael Krawczak, Arne Lindgren, Hugh S. Markus, Olle Melander, Mayowa Owolabi, Kristina Schlicht, Martin Söderholm, Vinodh Srinivasasainagendra, Carolina Soriano Tárraga, Martin Stenman, Hemant Tiwari, Margaret Corasaniti, Natalie Fecteau, Beth Guizzardi, Haley Lopez, Kevin Nguyen, Brady Gaynor, Timothy O’Connor, O. Colin Stine, Steven J. Kittner, Patrick McArdle, Braxton D. Mitchell, Huichun Xu, Caspar Grond-Ginsbach

**Affiliations:** 1 Veterans Affairs Maryland Health Care System, University of Maryland School of Medicine, Baltimore, MD, United States of America; 2 University of Ibadan, Ibadan, Nigeria; 3 Unversity of Cambridge, Cambridge, England, United Kingdom; 4 National Center for Cardiovascular Diseases, Beijing, China; 5 IMIM-Hospital del Mar; Universitat Autònoma de Barcelona, Barcelona, Spain; 6 Institute of Research Hospital de la Santa Creu I Sant Pau, Barcelona, Spain; 7 University of Texas Health Science at Houston, Institute of Molecular Medicine & School of Public Health, Houston, TX, United States of America; 8 University of Gothenburg, Gothenburg, Sweden; 9 Institute of Medical Statistics and Informatics, University of Kiel, Kiel, Germany; 10 Neurology, Lund University, Lund, Sweden; 11 Department of Clinical Sciences Malmö, Lund University, Lund, Sweden; 12 Department of Neurology, Skåne University Hospital Malmö and Lund, Lund, Sweden; 13 School of Public Health, University of Alabama at Birmingham, Birmingham, AL, United States of America; 14 Lund University, Lund, Sweden; 15 University of Maryland School of Medicine, Baltimore, MD, United States of America; 16 Heidelberg University, Heidelberg, Gemany; PLOS ONE, UNITED STATES

## Abstract

**Background and purpose:**

The role of copy number variation (CNV) variation in stroke susceptibility and outcome has yet to be explored. The Copy Number Variation and Stroke (CaNVAS) Risk and Outcome study addresses this knowledge gap.

**Methods:**

Over 24,500 well-phenotyped IS cases, including IS subtypes, and over 43,500 controls have been identified, all with readily available genotyping on GWAS and exome arrays, with case measures of stroke outcome. To evaluate CNV-associated stroke risk and stroke outcome it is planned to: 1) perform Risk Discovery using several analytic approaches to identify CNVs that are associated with the risk of IS and its subtypes, across the age-, sex- and ethnicity-spectrums; 2) perform Risk Replication and Extension to determine whether the identified stroke-associated CNVs replicate in other ethnically diverse datasets and use biomarker data (e.g. methylation, proteomic, RNA, miRNA, etc.) to evaluate how the identified CNVs exert their effects on stroke risk, and lastly; 3) perform outcome-based Replication and Extension analyses of recent findings demonstrating an inverse relationship between CNV burden and stroke outcome at 3 months (mRS), and then determine the key CNV drivers responsible for these associations using existing biomarker data.

**Results:**

The results of an initial CNV evaluation of 50 samples from each participating dataset are presented demonstrating that the existing GWAS and exome chip data are excellent for the planned CNV analyses. Further, some samples will require additional considerations for analysis, however such samples can readily be identified, as demonstrated by a sample demonstrating clonal mosaicism.

**Conclusion:**

The CaNVAS study will cost-effectively leverage the numerous advantages of using existing case-control data sets, exploring the relationships between CNV and IS and its subtypes, and outcome at 3 months, in both men and women, in those of African and European-Caucasian descent, this, across the entire adult-age spectrum.

## Introduction

In the United States, stroke is the leading cause of serious long-term disability and the 4th leading cause of death [1, 2]. In contrast to myocardial infarction, where the underlying pathology is almost exclusively atherosclerotic, large artery atherosclerosis accounts only for 12% of ischemic stroke (IS) incidence [3]. The etiology of IS complex, and understanding its pathophysiology can aid in prevention and improve treatment. As for many other complex diseases, one approach to understanding etiology is genetics, which can identify novel pathways and drug targets through an unbiased approach.

Although large genome-wide association studies (GWAS) of ischemic stroke (IS) populations have been successful at identifying stroke-associated loci with small effect sizes, copy number variation (CNV) analyses of these same datasets has yet to occur. Studying CNV has revealed important insights for numerous other complex diseases and, in fact, our preliminary data demonstrates several CNV associations to biologically compelling ischemic stroke candidate loci. Moreover, we have recently demonstrated that a higher CNV burden genome-wide is associated with poorer stroke outcome at 3 months using the modified Rankin Scale (mRS) [4]. We therefore hypothesize that CNV analyses of existing GWAS and exome array data will be a highly effective and cost-efficient methodology to identify novel associations illuminating stroke mechanisms, treatment targets, and outcome drivers. We further speculate that these analyses of existing GWAS microarrays will also identify rare and *de novo* CNVs of large effect size in ischemic stroke, as suggested by the existence of numerous monogenic, syndromic and complex diseases associated with CNV. CNV studies therefore may bridge the gap between common SNPs associated with common stroke and rare mutations, causing familial stroke syndromes, thus partially explaining the ‘missing heritability’ known to exist in stroke.

### Prior studies on the heritability of stroke

Twin studies suggest a significant heritability for stroke. Monozygotic twins are more likely (odds ratio ∼2.0) to be concordant for stroke than dizygotic twins [5–7]. Other studies evaluating IS genetics across the age-spectrum demonstrated a stronger genetic contribution to early-onset stroke, serving as a motivator for the planned age-stratified CNV analyses. In these studies, it was demonstrated that a gradient of greater familial aggregation exists in younger cases [8], that extended into the young-adult age range [9]. Consistent with these findings, and since familial aggregation can also be due to shared environmental influences, genetic heritability analyses based on common variant GWAS data showed that IS cases younger than 55 years of age had higher heritability compared to older IS cases (42% ±8%, P < 0.001 versus 34% ±10%, P < 0.001) [10].

Specific to the CaNVAS study, the heritability of stroke has previously been evaluated in the African-ancestry South London Ethnicity and Stroke Study (SLESS) population (included in CaNVAS) using GREML (genomic-relatedness matrix-restricted maximum likelihood) approaches [11]. Based on sample size limitations, restricted analyses to the phenotype of all-stroke vs. controls, including 10 ancestry-informative principal components to control for population structure, estimated the population prevalence of stroke in England at 2.3%. Implementing a relatedness threshold of 0.05 (equivalent to second-cousin relatedness), 161 individuals were removed (89 cases, 72 controls). Ultimately, a genetic contribution to IS in SLESS (p = 0.043) was found with an estimated heritability of 0.35 (SE 0.19). If the prevalence were assumed to be higher (4%), this estimate would rise to 0.41 (SE 0.23); whereas for a lower prevalence (1%), the estimate is 0.26 (SE 0.16). This compares to a heritability estimate of 0.18 in the largest analysis in Europeans to date [12].

There are other studies evaluating stroke-subtype heritability. First, using single nucleotide polymorphism (SNP)-based pseudo-heritability measures heritability estimates in cardioembolic stroke were demonstrated increasing from 16.5% in older onset cases to 28.5% in younger onset cases [13]. Two other studies [12, 14] reporting GWAS-derived heritability measures using GCTA software [15] demonstrated variability in the heritability estimates. Both studies agreed on the heritability estimate for stroke as a whole of ∼40%. However, heritability estimates by stroke subtype varied markedly. The large vessel subtype always showed the highest heritability measures (40.3 and 66%) while the small vessel subtype showed the lowest measures (16.1 and 10%), although accuarate phenotyping may play a role, as higher heritabilities were seen in magnetic resonance imaging–verified lacunar stroke (20%–25%) [16].

### Missing heritability

While the described findings demonstrate a strong heritabilitable component for ischemic stroke, which is enriched in early-onset cases, it is important to note that the total contribution of all identified and replicated genetic stroke risk factors [17] remains far below the estimated stroke heritability measures as described. Hence, with few genome-wide loci for stroke identified thus far, there remains a substantial proportion of missing heritability, with CNV as a thus far unexamined potential contributor.

### CNV and stroke risk

A relatively recent review, as summarized in **Table 1**, highlights the prior CNV findings in patients with ischemic stroke [18] emphasizing rare CNVs causing Mendelian stroke syndromes, common CNVs associated with stroke risk factors, and CNV associated with particular stroke subtypes, including cervical artery dissection, small vessel disease or Moyamoya disease.

**Table 1:** CNV-findings associated with ischemic stroke.

Phenotype	CNV	affected/disrupted genes	Ref
CNV-findings in stroke due to a Mendelian disorder			
CADASIL	100 bp deletion	*NOTCH3*	[19]
Vascular EDS	2q32 deletion	*COL3A1*, *COL5A2*	[20]
CNV associated with subtypes of ischemic stroke			
CeAD	erichment of various CNVs affecting arterial development		[21]
CeAD	16p13.1 duplication	*MYH11/ABCC6 locus*	[21]
Moya-moya	6pter duplications		[22, 23]
CCM	exonic CNVs	*CCM1; CCM2; CCM3*	[24]
SAO	13q34 duplication	*COL4A1/COL4A2 locus*	[25, 26]
SAO	low (<4) copy number	*DEFB4*	[27]
LVD	low (<4) copy number	*DEFB4*	[27]
CNV associated with complex developmental retardation phenotypes and pediatric stroke			
	1q24 /10q26 deletions	*SERPINC1*	[28]
CNV associated with stroke risk factors			
Atrial fibrillation	intronic duplication	*KCNIP1*	[29]
Obesity	CNV burden		[30]
Obesity	16p11.2/22q11.2 deletion		[31]
Obesity	low copy number	*AMY1*	[32]
Hyperlipidemia	VNTR	*LDLR*, *LPA*	[33]

CADASIL: Cerebral Autosomal Dominant Arteriopathy with Subcortical Infarcts and Leukoencephalopathy; EDS: Ehlers Danlos syndrome; SAO: Small arterial occlusive disease; LVD: Large vessel disease; CeAD: Cervical artery dissection; CCM: Cerebral cavernous malformations; VNTR: Variable number of tandem repeats.

In these early subtype-specific CNV studies, 70 CeAD patients were phenotyped by an electron-microscopic analysis of a skin biopsy in order to detect connective tissue alterations [20]. One patient with carotid artery dissection and a history of aortic disease had a large deletion covering the entirety of the *COL3A1* and *COL5A2* genes [20]. Another patient carried a large recurrent duplication of chromosome 16p13 including the *MYH11* and *ABCC6* genes, a rare finding in the normal population that predisposes to aortic aneurysm and dissection [34, 35]. Four further patients with CNV of the *MYH11/ABCC6* locus were identified in a subsequent exploration of 833 CeAD patients [21]. Interestingly, this latter CNV-study of CeAD did not detect association with variation in a particular locus but found association with variation in a pre-defined set of genes involved in cardiovascular system development. To date, only a few small, underpowered studies [36] have evaluated CNV in the setting of IS.

### CNV and stroke outcome

Few studies exist evaluating the association between CNV and ischemic stroke outcome. One recent study demonstrated that genetic imbalance level (i.e. total CNV burden) was negatively associated with favorable outcome after IS [4]. CNV was identified in high-density SNP microarray data of IS patients from the Cervical Artery Dissection and Ischemic Stroke Patients (CADISP [37]), Stroke Genetics Network–NINDS (SiGN [38]) and Genetics of Ischaemic Stroke Functional Outcome (GISCOME [39]) networks. Genetic imbalance, defined as the total number of protein-coding genes affected by CNVs in an individual, was compared between patients with favorable (modified Rankin Scale, mRS = 0–2) and unfavorable (mRS >3) outcome after 3 months. Subgroup analyses were carried out confined to CNVs either affecting ohnologs, a class of dose-sensitive genes, or not.

**Note on ohnologs**

The geneticist Susumu Ohno hypothesized that the large vertebrate genome developed from smaller primitive fish genomes by two rounds of whole genome duplications. As a consequence, many genes have four similar copies across the genome (for instance NOTCH1, NOTCH2, NOTCH3, and NOTCH2NL). Apparently, these copies were not redundant and have been evolutionary conserved over billions of years. Other copies, however, evolved into new functions (“neo-functionalization) and have no detectable homologous relatives within the genome. Although the evolutionary conserved genes (ohnologs) were originally present in multiple copies (after the initial whole genome duplication) all copies were apparently needed. This might indicate that these genes are particularly dose-sensitive, which was confirmed in other recent CNV studies: many disease-causing CNV include ohnologs [40, 41].

The association of imbalance with outcome was analyzed by logistic regression analysis, adjusted for age, sex, stroke subtype, stroke severity and ancestry. The study sample comprised 816 CADISP patients (age 44.2±10.3 years) and 2498 SiGN/GISCOME patients (age 67.7±14.2 years). Outcome was unfavorable in 122 CADISP and in 889 SiGN/GISCOME patients. Multivariate logistic regression analysis revealed that imbalance was negatively associated with favorable outcome in both samples (CADISP: p = 0.0007; OR (odds ratio): 0.89; 95% confidence interval (95%CI): 0.82–0.95; SiGN/Giscome: p = 0.0036, OR:0.94; 95%CI:0.91–0.98). The association was independent of age, sex, stroke severity upon admission, stroke subtype and ancestry. In our study, upon subgroup analysis, imbalance affecting *ohnologs* was associated with outcome in both study populations (CADISP: OR: 0.88; 95%CI: 0.80–0.95; SiGN/Giscome: OR: 0.93; 95%CI: 0.89–0.9) whereas imbalance *without ohnologs* lacked such an association. From these subgroup analyses we concluded that the identified associations were driven by the presence of ohnologs in the respective CNVs, suggesting a truly causal role of the deleterious effects of genetic imbalance.

Overall, these described studies, demonstrate the scientific motivation and methodological basis for the Copy Number Variation and Stroke (CaNVAS) Risk and Outcome Study.

## Materials and methods

In the field of stroke genetics, the CaNVAS study is innovative for several reasons:

### 1. Patients

The study sample includes large African ancestry cohorts, in addition to large European-Caucasian cohorts, with sample sizes well powered to evaluate ischemic stroke subtypes, sex differences, across the age-spectrum [42, 43].

### 2. Type of genetic variation

Focus on CNV, and in particular on genomic imbalance, as a methodology to identify key CNV drivers, genes and pathways related to stroke risk and outcome.

**Note on genetic variation**

Human genetic variation can be classified with regard to frequency (rare or common variants), function (pathogenic, benign or variants of unknown significance–VUS) or size (single nucleotide polymorphisms (SNPs), microsatellites (oligo-nucleotide repeats), indels, copy number variants–CNVs, or aneuploidy). CNVs are usually defined as structural variants >100 base-pairs of DNA. CNV typically map in genomic regions that are rich in repeated sequences (segmental duplications) and have a higher rate of new mutation than SNPs.

Most current studies in stroke genetics relate to either rare pathogenic variants associated with rare Mendelian disorders (like CADASIL or Ehlers-Danlos syndrome) or common SNPs with small effects sizes. Notably, the CaNVAS study will address the intermediate class of structural variation including CNV which have not been systematically explored in IS.

### 3. Innovative CNV analysis methodology

CaNVAS is both cost-effective and immediate, utilizing pre-existing GWAS microarray data from well characterized patient cohorts and from controls. Further, high-quality CNV identification methods will be employed implementing: a) software-assisted noise reduction; b) quality control including detection of clonal mosaicism, and; c) analysis of long runs of homozygosity to assess degree of consanguinity.

### 4. Includes analysis of outcome / recovery after ischemic stroke

In additional datasets, one goal is to replicate recent findings demonstrating that an increased CNV burden is associated with worse outcome at 3-months post-stroke [4], and then using all datasets in CaNVAS perform analyses to identify the key CNV drivers, genes and pathways responsible for these relationships.

### 5. Investigations of CNV function

As to be discussed, CNV associated with stroke risk and outcome will be assessed for functionality using *existing* biomarker data from the TOPMed and GeneStroke Cconsortiums.

### 6. Creation of a new junior investigator training network

Given the international structure of the CaNVAS Study and a desire to promote consistent scientific involvement across all sites, a training network was developed within the CaNVAS study for Ph.D. Students and Post-Doctoral Research Fellows. These genetics trainees will be supervised by their respective CaNVAS site PI and tasked with site-specific responsibilities related to the project. They will receive CNV methodological training, and participate in all phases of the study, including monthly study and trainee web-based conference calls. Senior CaNVAS investigators will ‘rotate’ providing monthly lectures and seminars for the trainees.

### Study subjects

CaNVAS study subjects and variables as related to each cohort are described in **Table 2**.

**Table 2:** Summary of CaNVAS study subjects and other cohort specific variables.

Study (reference)	Ancestry	Number of cases (Goals 1 and 3)	Number of controls (Goal 1)	Control source	GWAS chip	Exome chip data available	Outcome mRS @ 3 months (Goal 3)	Biomarker Data (Goals 2 and 3)
GEOS-USA [44]	CAU	448	498	Internal	Illumina 1M	Yes	No (100% w/ mRS at discharge)	No
GEOS-USA [44]	AFR	381	352	Internal	Illumina 1M	Yes	No (100% w/ mRS at discharge)	No
Krakow-Poland [38]	CAU	952	776	Internal	Illumina 5M	Yes	No (100% w/ mRS at discharge)	No
Leuven-Belgium [38]	CAU	469	468	Internal	Illumina 5M	Yes	Yes; n = 469	No
CADISP-European [37]	CAU	565	1260	Internal	Illumina 610K or 670K	No	Yes; n = 565	No
South Swedish GWAS Study (Partial SiGN) [38]	CAU	3500 (SiGN: 1500; non-SiGN: 2000)	5500	Internal	SiGN: Illumina 5M	Yes	Yes, ~40%; n = 1400 (100% w/ mRS at discharge)	No
					Non-SiGN: OmniExpressExome BeadChip V1.1			
GeneStroke: Sant Pau-Spain [45]	CAU	2571	505	Internal	Illumina 5 M and Illumina Human Core Exome	Yes	Yes; n = 2571	Yes Epigenetic (n = ~300), 52 cases / controls with proteomic data. 230 cases / controls with epigenetic data.
GeneStroke: IMIM-Spain (Partial SiGN) [38]	CAU	2709 (SiGN:1035; non-SiGN: 1674)	1000	Internal	SiGN:Illumina 5M	Yes	Yes; n = 2709	Yes (case data only; control data pending) Epigenetic (n = 1072). miRNA (n = 260). RNAs (n = 127; 40 samples at three timepoints: 6hr, 24hr and 3 months post-stroke).
					Non-SiGN: Illumina Omni2.5, CoreExome 12			
SLESS–UK [11]	AFR	808	868	Internal	Illumina 1.7M Multi-Ethnic	No	No	Yes (e.g. Homocysteine, coagulation factors)
SIREN–Nigeria [43]	AFR	1700	1700	Internal	Illumina 2.5M H3Africa	No	Yes, ~50%; n = 850	No
Additional SiGN [38]	CAU/AFR	5765 (942 Hisp)	0	External	Illumina 5M	Yes	Yes, ~50%; n = 2882	No
Health and Retirement Study [46]	CAU/AFR	0	11724 (1136 Hisp)	N/A	Illumina 2.5M	Yes	N/A	No
**Total**		**19868**	**24651**				**11446**	
**Replication and Lookup (Goal 2)**								
TOPMed (WHI/FHS/JHS/ ARIC/MESA) [47]	CAU/AFR	4665 (151 Hisp)	19283 (1105 Hisp)	Internal	Whole Genome Sequencing (WGS)	Yes, WGS	No	Yes, many
**Cumulative Tota**l		**24533**	**43934**					

### Data management

A centralized data repository is being created at the University of Maryland (UMD) Baltimore with analyses occuring at several sites. Notably, the UMD served as the Data Management Core for the SiGN Consortium [38, 48, 49], organizing the transfer of DNA, genotype and phenotype data from/to each study site and then ensuring harmonization of required covariate data. UMD will utilize pre-existing procedures and pipelines for transfer of data, checking of variables for missing and out of bounds values, and harmonization as needed. Similar procedures are in place at other analyses sites and will be harmonized across all sites. Secured access will be provided to the data for authorized personnel only. Reproducibility of research findings is of growing concern to the larger scientific community and clinical research in particular. Reproducibility will be maintained through the use of project specific git-repository (version tracking software). Due to the large volume of data associated with CaNVAS, data redundancy will be minimized, with duplicate data files constructed only when analytic processing requires. Notably, all international data limitations regarding genetic and phenotype data transfers will be respected.

### Stroke phenotyping

All included studies have datasets with previously assigned ischemic stroke subtypes using the TOAST subtype classification system [50] and/or the CCS-Causative Classification System [51], categorizing stroke cases on the basis of the presumed mechanism: large-artery atherosclerosis, cardioembolism, small-artery occlusion, other known etiology, and undetermined etiology. Analyses evaluating both classification systems when available will be employed. Notably, most replicated GWAS loci identified in IS have been subtype-specific [17], which is also consistent with the CNV findings as listed in **Table 1**. With the large sample size of the proposed study, CaNVAS is expected to have an enhanced ability to examine subtype specificity.

### Genome–wide CNV identification

All data as required for the CNV analyses are readily available from the prior SNP microarrays. Phenotype data files will be maintained with SAS (v9.4), and scripts written for transfer to multiple formats. The genotype data (GWAS) needed for principal components will be kept in text and PLINK formats.

**Note on CNV detection**

CNV can be identified on a genome-wide scale in next generation sequencing data and in high-density SNP microarrays. CaNVAS reanalyzes pre-existing GWAS microarrays, which is an excellent platform for the study of larger (>10 kilobases of DNA) CNV. For CNV detection automatic established software algorithms will be used. Using software to reduce noise in GWAS microarrays [52] there will be improved exclusion of false positive CNV findings. Precise breakpoint estimates of CNV-findings enables mapping on the human genome, assessment of genetic content, and comparison with established databases.

### Quality control and identification

All participating studies have previously been genotyped on high-density SNP microarray platforms. Many of the participating studies have internal controls that were genotyped alongside the cases. Previously utilized external controls (n = 11,724) from the Health and Retirement Study (HRS) will be used for the SiGN cases without internal controls. In addition to the SiGN data, the UMD and other analyses sites will work together to attain and transfer the necessary data files for the CNV analyses from each participating site. The ability to implement PennCNV at all sites has been confirmed, as this will be necessary for the junior trainee participatory investigations. All sites have IRB approval for the studies and collaborations as proposed; formal IRB approval for this particular study will be attained at each site. All investigators and junior trainees will attain appropriate human subjects training.

SiGN sample-level quality control steps will be implemented on all samples including removal of study samples exhibiting mismatches between genetic and reported sex, and those appearing as outliers from the population on the basis of principal component analysis of SNPs. Tests for cryptic relatedness will be performed to exclude related samples to avoid inflation of test statistics. Population stratification will be accounted for in a variety of ways as consistent with recent CNV analyses of stroke outcome [4]. Specific CNV QC measures include identification of samples with clonal mosaicism (a condition occurring in >3% of humans older than 80 years), with outlier number of CNV calls and with variance of signal intensity values (LRR) >0.2 as described previously [4].

Microarrays for each study population will be analyzed using automatic CNV detection algorithms including PennCNV [53], and at least one further software package like QuantiSNP [54] or DNAcopy [55]. Data transfer, automatic CNV-detection and filtering of CNV-findings with regards to size (number of SNPs or physical length in base-pairs) and to genetic content (exclusion of CNV-findings without coding sequences) will be performed using standardized protocols. Eligible CNV findings of each individual dataset will be manually inspected after noise reduction with the noise-free-CNV software [52] to identify and exclude false positive findings. Since a large part of the noise in SNP microarrays is systematic, comparison of target samples with referent samples will enable significant noise-reduction. As consistent with standard in Comparative Genomic Hybridization methods, the noise-free-CNV software introduces similar pairwise comparative approaches into the field of SNP microarray analyses.

### CNV mapping and functional classification

Confirmed CNV-findings will be characterized by mapping their breakpoints (start-SNP; end-SNP) onto the human genome [56] to assess the size (physical length of the DNA sequence) of the CNV; to define the CNV-finding as genic (affecting protein-coding genes) or non-genic CNVs (located in introns, in intergenic regions or affecting non-coding transcripts) and to determine the number of protein-coding genes affected by the CNV. Comparison with findings from other patients, controls or public databases allows the classification of the CNV-finding as unique versus recurrent, as rare (minor allele frequency <1%) versus common, and as complex rearrangements versus simple CNV.

The genetic content of the CNV-findings may indicate its functional impact. Large CNV covering several whole genes are more likely to be deleterious than small CNV findings, deletions (loss of functional genes) may have a stronger phenotype than duplications (gain of genetic material). Because ohnologs were recently identified as a class of dose-sensitive genes, all genes affected by CNV will be classified accordingly by searching in the Ohnolog Repository [40]. CNV affecting genes that are known to cause Mendelian disorders associated with stroke or that affect stroke risk factors (blood lipid level, blood pressure) are also potentially functional, when identified more frequently in patients than in controls. Established stroke loci will also be considered.

In general, it is important to note that functional annotation of CNV differs from functional annotation of coding SNPs, since most CNV do not result in missense, non-sense or loss-of-function variants. CNV do not necessarily result in up- or down-regulation of transcriptional activity, although they can. Since most genic CNVs are rare and since many different low-frequency genic CNVs occur, previously performed GWAS SNP-based case-control studies may be underpowered to detect disease-association.

**Note on functional enrichment analysis**

Systematic study for association of CNV with stroke risk or with stroke outcome will make use of functional enrichment analysis (FEA) with public browsers like Database for Annotation, Visualization and Integrated Discovery (DAVID [57]), the generic Gene Ontology (GO) term finder [58], or others. Essential for valid FEA is the analysis of control samples: Association with a predefined gene group (GO term) or pathway is established if such enrichment is significant (after stringent correction for multiple testing) in the cases, but absent from the controls (or observed in the patients with poor outcome, but absent from the patients with favorable outcome). For an example of comparative FEA see a relatively recent CNV study of cervical artery dissection [21].

### Statistical methods

Statistical analysis of confirmed CNVs and stroke phenotypes will be performed mainly in R and SPSS. Datasheets and results may be converted to SPSS format if necessary to facilitate exchange with research partners and junior participants. Age, sex and phenotypic covariates like stroke subtype and vascular risk factors will be accounted for as appropriate.

### Goal 1. Risk Discovery

Using manually-curated and standard CNV analytic approaches, identify CNVs that are associated with the risk of IS and its subtypes, in over 19,500 cases from the SiGN Consortium (African and Caucasian ancestry), the SIREN Consortium (African ancestry), the South London Ethnicity and Stroke Study (SLESS) (African ancestry), the CADISP Consortium (Caucasian ancestry), GeneStroke Consortium (Caucasian ancestry), South Sweden Study (Caucasian ancestry) and in controls.

### Motivation

Goal 1 is to identify CNV associated with IS using available data from available GWAS and exome arrays. The numerous participating studies brought together by CaNVAS are described in **Table 2** and comprise over 19,500 cases and over 24,500 controls with GWAS data, and exome content in well over 50%. The selected CaNVAS studies were chosen to balance subjects of African and Caucasian ancestry, as well as age and sex. This risk goal will utilize a case-control design, with the sources of controls for each study also provided in **Table 2.** Internal control availability was considered, as was the availability of case outcome data as required for Goal 3. Notably, through prior participation in the SiGN Consortium [48], the CaNVAS investigators have extensive experience utilizing external controls to ensure appropriate case/control matching [38]. Available Hispanic data (as indicated in **Table 2**) will be analyzed, although power will be limited.

### Single-CNV risk analyses

Classical methods of genetic association analysis, including logistic regression modelling, will be used to evaluate the impact of CNV genotype on stroke risk, both in single CNV-based and in pathway-based approaches (Goals 1 and 2). Analyses will be stratified by stroke subtype, age, sex or other covariates, depending upon data structure. We plan to run single CNV association analyses for those with minor allele count (MAC) > 10 as well as implementing binning approaches for less frequent but regionally localized CNVs.

### Single-CNV power

The stroke risk assessments of Goals 1 and 2 form the primary endpoint for all power and sample size estimations in this study. There is little information on what constitutes genome-wide significance in CNV studies of this type. The first CaNVAS hypothesis is that specific CNVs are associated with stroke risk. In this situation, at an alpha level of 5 X 10^−08^, hence the sample of >19,000 stroke cases should provide 80% power to detect CNVs with ORs ranging from 1.10 to 1.22 across CNV frequencies ranging from 5% to 50%.

Investigations akin to Girirajan et al. [59] will also be performed, in which a total of 120 genomic regions potentially prone to recurrent CNVs were identified because they are flanked by segments of high homology, called segmental duplications. CaNVAS subjects will similarly be evaluated to determine if stroke cases are more likely than controls to have regions of segmental duplications as based on the 120 regions previously identified [59]. Since particular regions will be explored, a Bonferroni correction for multiple testing of recurrent CNVs (those flanked by segmental duplications) might require a p-value of <4.1×10^−4^ to be accepted as a significant association for this particular type of CNV (p = 0.05/120). Based upon the large CaNVAS sample size, our power will be high for such tests.

### Functional annotation

Once associated CNVs have been identified, functional annotation merging with the identified CNV loci with other known stroke susceptibility loci will occur. Basic annotation, such as for evaluating known missense and loss of function variants with the identified CNV, can be integrated using Variant Effect Predictor (VEP [60]). Overlap between CNV findings and prior risk variants might imply risk mechanisms. Further, it is possible that some study participants with CNV may have been excluded from GWAS SNP-based analyses as allelic frequencies would not be in HWE. VEP includes ‘LOFTEE,’ an additional tool for filtering loss of function variants (which are particularly challenging to annotate). Publicly available Hi-C data, which measures the 3-dimensional folding of the genome, can also be used to find genomic regions that interact and might be disrupted in the setting of CNV. Additionally, since many credible CNV may not lie within a gene, functional annotations from ENSEMBL, ENCODE and ROADMAP will be ‘layered-in’, annotating all CNVs in this set to prioritize those most likely to be functional. The GTEx resource together with transcriptome wide association analysis (see below) we also be utilized to evaluate whether CNVs in this set alter gene expression in various tissue sets.

### Integration of transcriptome association mapping

Once credible sets of causal CNV have been identified, they will be leveraged to try to identify the causal genes, as well as ‘dig deeper’ to identify the most likely causal CNV within each set employing functional annotation and integrating predicted transcriptome data with the CNV results. Prediction of gene expression is based on the fact that gene expression can be portioned into a component that is genetically determined and a component that is environmentally determined (e.g., disease state causes change in gene expression). The PredixScan software tool allows one to predict the genetically determined component of gene expression by using models developed across a range of tissues and available through public resources (e.g., GTEx) [61]. From these models, one can use the observed CNVs in one’s dataset to predict tissue-specific genome-wide gene expression. This approach will be employed to determine which (if any) genes in associated regions are predicted to be differentially expressed between cases and controls. Because this method estimates only the genetically predicted component of gene expression, it is suitable for predicting genes likely to be causal in disease.

### Gene-based risk then pathway-based risk analyses

At this point in the CaNVAS analyses, single-CNV and gene-burden CNV results will be available. Next, implementing a unbiased hierarchical clustering on gene-level data (after removing non-altered genes to reduce the visualization complexity), clusters will be evaluated to determine if phenotype categories (e.g. stroke subtypes, sex, age<50, among others) demonstrate differential results. To apply this on a pathway level, data will be collapsed to pathways (group genes into pathways) as based on KEGG pathway database, Cytoscape, Ingenuity Pathway Analysis, Biocarta, and Reactome databases which reflect metabolic, biochemical and signaling processes, and then perform clustering analyses based upon these pathways.

### Gene-based risk then pathway-based power

Regarding associations with individual genes, assuming all 20,000 protein-coding genes are tested, a conservative Bonferroni correction would require a p<2.5×10^−6^ (p = 0.05/20,000). However, collapsing CNV-findings on "biological processes", "predefined gene groups" (Gene Ontology bases) and "pathways" liberalizes p-value significance requirements. For example, analyzing all CNVs disrupting inflammatory response or those in the TGF beta-receptor signalling pathway or those related to a cell type or structure. As reported by Grond-Ginsbach et al. [21], significant CNV associations were found with arterial connective tissue structure, with significant findings of the functional enrichment analyses after correction for multiple testing, this, in a much smaller sample size (833 CeAD patients and 2040 control subjects) than that available in CaNVAS. Single CNV analysis may be performed for selected CNVs, for instance as similar to the large *MYH11/ABCC6* CNV in **Table 1** [21], but not on a genome-wide level. Hence, for stroke subtype it is estimated that ~800 patients and ~2000 controls are required. However, since heritability of dissection may be higher and heterogeneity less than for other stroke subtypes, larger numbers are preferred, for instance ~2000 or more patients for each subtype. Such numbers are readily available in the CaNVAS study population.

At the completion of these stroke risk-related analyses a list of ‘top’ CNVs, genes and pathways will be generated that will undergo the replication and biomarker evaluations in the TOPMed cohorts (Goal 2).

### Goal 2. Risk replication and extension

Determine whether the CNVs associated with IS in Goal 1 are also associated with IS in the TopMed Consortia (African and Caucasian ancestry), and then evaluate to what extent the identified CNVs exert their effects on stroke risk via their effects on stroke risk factors that have also been measured in TOPMed (e.g., blood pressure, circulating biomarkers of inflammation and coagulation, blood miRNA, mRNA, methylation, metabolomics, and others). Similar analyses will be performed using existing GeneStroke Consortium (Caucasian ancestry) biomarker data.

### Motivation

The intention here is to replicate CNV findings as identified in Goal 1 using TOPMed WGS data. Then use available TOPMed an other biomarker data to determine the mechanisms of action of the identified risk associated CNVs.

### Replication

Multiple approaches will be employed to follow-up associations detected in Goal 1 for the purpose of identifying causal CNVs, genes, and pathways associated with IS. First, identified CNVs from Goal 1 will be evaluated for replication in existing TOPMed datasets. As indicated in **Table 2**, the TOPMED datasets includes 4,665 IS and 19,283 controls samples of primarily Caucasian and African descent. The majority of TOPMed stroke patients have undergone stroke subtyping with contributing studies including: the Women’s Health Initiative (WHI: African/Caucasian/Hispanic), the Framingham Heart Study (FHS: Caucasian), Jackson Heart Study (JHS: African), the Atherosclerosis Risk in Communities Study (ARIC: African/Caucasian), and the Multi-Ethnic Study of Atherosclerosis (MESA: African/Caucasian/Hispanic) ensuring adequate ethnically-diverse populations for our replication efforts. All TOPMed subjects have undergone WGS and/or GWAS. Using these data, IS associated CNVs can easily be replicated, and evaluated according to multiple criteria, including strength of association across subtype, sex, ethnicity, age, vascular risk factors, as considered in the setting of plausible stroke mechanisms, among other considerations. These risk replication analyses will include singe-CNV, gene-based and pathway-based replication analyses as consistent with those identified in Goal 1.

### Extension

Based upon the Goal 2 replication results, biomarker evaluations as stratified by CNV will be employed using data from TOPMed and other cohorts; the goal of these evaluations is to identify measurable biomarker differences relating to stroke risk mechanisms. As examples, potential biomarkers can include all previously attained measures available in the TOPMed data sets, such as blood pressure, circulating biomarkers of inflammation and coagulation, blood miRNA, mRNA, methylation, metabolomics, among others. Here, TOPMed participants (stroke and non-stroke) can be stratified by the identified CNVs frequency or size to evaluate if a specific biomarker correlates with that copy number variant. While determining the CNV-mediated relationships between stroke risk and the biomarker is the goal, non-stroke controls subject biomarker levels will be used as baseline measures, as some biomarkers will likely change in the presence of stroke. In the stroke patients, given the cohort nature of the TOPMed studies, pre-stroke biomarker data will also be evaluated and contrasted with post-stroke measures. While the Goal 1 results will guide which subjects should be evaluated on the basis of ethnicity, sex and age, available biomarker data is a limitation (discussed below).

As an example of a potential biomarker CNV-stratified comparison, consider Goal 1 identifies and Goal 2 replicates a stroke associated intronic CNV of uncertain significance with a greater frequency in hypertensive blacks than whites. Strata based on the presence or absence of the CNV, then evaluating available biomarkers (+/- hypertension (HTN), creatinine levels, measured known gene products related to HTN, etc.) would be created. These might include; 1) All non-stroke participants by sex and ethnicity to determine baseline measures; 2) Further stratified by HTN; followed by stratified comparisons of 3) Specific-stroke subtypes vs. non-strokes.

Beyond standard vascular risk factors (HTN, diabetes, smoking status, etc.), numerous circulating biomarkers are available in the TOPMed data (**Table 3**), as are miRNA-whole blood and extracellular, mRNA, methylation, 80 proteins, SomaLogic Proteomics, and ~450 metabolomics markers, all of which can be explored. Notably, in the Women’s Health Initiative, a participating TOPMed study, the Proseek Multiplex CVD III panel (see reference link: Proseek [62]) of 92 cardiovascular protein biomarkers are being measured in baseline blood samples in 2,000 or more participants. This panel includes 6 proteins with central roles in coagulation (tissue-type plasminogen activator (t-PA), plasminogen activator inhibitor 1 (PAI), urokinase-type plasminogen activator (uPA), urokinase plasminogen activator surface receptor (U-PAR), tissue factor pathway inhibitor (TFPI), and von Willebrand factor (vWF)). Many of the other biomarkers on this panel play a role in the immune system or inflammation and are relevant because of the tight stroke-related biological link between inflammation and coagulation pathways [63, 64]. Lastly, the GeneStroke Consortium included in CaNVAS also has similar existing biomarker data (e.g. methylation, proteomic, RNA, miRNA) in a subset of the CaNVAS cases and controls, thereby allowing for direct comparisons within the same subjects, and providing further replication for the TOPMed results and vice-versa.

**Table 3:** Partial list of available biomarkers in TOPMed.

Circulating biomarkers	
APOE	E2, E3, and E4 genotype, and circulating APOE4 levels
Polyunsaturated fatty acids	Docosahexaenoic acid (DHA), total omega-3 fatty acids, other RBC membrane fatty acids
Inflammation	C-reactive protein (CRP), interleukin-6 (IL-6), intracellular adhesion molecule (ICAM-1), myeloperoxidase, osteoprotegerin, P-selectin CD40 ligand, monocyte chemoattractant protein-1 (MCP- 1), TNF-alpha and its receptor TNF-R22 and lP-PLA2
Hemostasis and thrombosis	Fibrinogen, Factor VIIIc, von Willebrand factor, D-dimer, PA I-1
Lipid metabolism	Total cholesterol, LDL, HDL, Apolipoprotein A1, B100 and Lipoprotein (a)
Molecules interacting with vessel wall and platelets	Markers of matrix remodeling (MMP-9, MMP-3, TIMP-1), plasma homocysteine, asymmetric dimethylarginine (ADMA)
Oxidative stress	Isoprostanes (IsoPs), uric acid
Hormones	Renin-angiotensin-aldosterone pathway, measures of thyroid function (e.g. TSH), sex steroid hormones, natriuretic pathway peptides (including BNP, NT, ANP)
Vitamins	Folate, un metabolized folate, B12, B6, vitamin D
Growth factors and their receptors	IGF-I, VEGF, BDNF, NGF
Homocysteine	Homocysteine, post methionine load homocysteine, MMA
Adipokines	Leptin, leptin receptor, alpha fetuin, Ghrelin, retinal binding protein 4 (RBP4), adipocyte fatty acid binding protein (A- FABPR), adiponectin
Glycemic control and insulin resistance	Hemoglobin A1C, Fasting and Postprandial blood sugar, categorization as impaired fasting glucose (IGF), impaired glucose tolerance (IGT), fasting and post-prandial insulin levels, measures of insulin resistance (H0MA-IR, Insulin sensitivity index (ISI 0–120))
Markers of renal injury	Cystatin-C, urine microalbumin
Markers of myocardial injury	Troponin I, GDF-15, ST-2
Markers of brain injury	S-100b, NSE (Neuron-specific enolase), GFAP (glial fibrillary acidic protein marker of glial injury)
Putative AD markers	Plasma Aβ measurements, Clusterin, Tau, Amylin

In summary, evaluating how risk-associated CNV modifies measurable biomarkers will infer on stroke prevention and treatment strategies.

### Goal 3. Stroke outcome replication and extension

Using manually-curated and standard CNV analytic approaches replicate recent findings demonstrating an inverse relationship between CNV burden and stroke outcome at 3 months (mRS) in over 8,100 *additional* cases from the SiGN Consortium (African and Caucasian ancestry), the CADISP Consortium (Caucasian ancestry), GeneStroke Consortium (Caucasian ancestry), the SIREN Consortium (African ancestry), and then using new and prior data determine the key CNV drivers responsible for these associations implementing gene- and pathway-based analyses, and by analyzing existing biomarker data (e.g. methylation, proteomic, RNA, miRNA) in the GeneStroke Consortium (Caucasian ancestry).

### Motivation

As described in the Preliminary Studies Section of this manuscript, a recent study demonstrated that genetic imbalance level (i.e. total CNV burden) was negatively associated with favorable outcome after IS [4]. These results form the basis of the CaNVAS Goal 3 efforts. First, using over 8,100 *additional* samples from SIGN, SIREN, SWEDEN and GeneStroke the Pfeiffer et al. [4] results will be replicated. Following these efforts, a combined dataset consisting of n = 3,314 [4] and the new n = 8,132 cases will be explored to determine which CNVs are the key drivers for these outcome relationships (total n = 11,446). Notably, the same cases used in Goal 1, are also used here, hence there is no further CNV calling required, rather only analyses based upon outcome.

### Single-CNV outcome (case-only) analyses

As seen in **Table 2,** under the column header ‘Outcome mRS @ 3 months’ (mRS = modified Rankin Scale), the cases with mRS data are listed. First, to replicate prior findings [4], new data will be evaluated for genetic imbalance (as defined as the total number of protein-coding genes affected by CNVs in an individual) as compared between patients with favorable (mRS = 0–2) and unfavorable (mRS >3) outcome after 3 months. Notably, several case data sets (see **Table 2.** GEOS, Krakow, SWEDEN—additional n = 3,881 cases) have mRS at time of stroke hospital discharge. Hence, a similar analyses using these mRS at discharge data sets will be performed, and pending results, can either be combined or meta-analyzed with our larger 3-month outcome datasets. Further analyses, that have not previously been performed, include evaluating mRS as a continuous variable and implementing shift analyses will also occur. Subgroup analyses will also be carried out confining CNVs to affecting ohnologs, or not. The association of imbalance with outcome will be analyzed by logistic regression analysis, adjusted for age, sex, stroke subtype, stroke severity (NIHSS) and ancestry. Variable-specific stratified analyses will also be performed pending results.

To this point, the methods employed have only evaluated for overall CNV burden. However, the goal is to identify individual CNVs of large effect, i.e. the key CNV outcome drivers. As such, classical methods of genetic association analysis, including logistic regression modelling, will be used to evaluate the impact of CNV genotype on stroke outcome, implementing single CNV-based and pathway-based approaches. Analyses will be stratified by stroke subtype, age, sex and other covariates, depending upon data structure. Possible confounding will be controlled for by the use of propensity scores. Statistical analysis will be performed mainly in R and SPSS. Thresholds for association analyses for single CNV association analyses will be limited to those with MAC > 10, as well as implementing a binning approach for less frequent but regionally localized CNVs.

### Single-CNV power

Consistent with the recent Pfeiffer et al. study [4], it is estimated that ~1/3 of the CaNVAS cases will have a poor outcome (n = 3777 (0.33 x 11,446)) and 2/3 will have a good outcome (n = 7669). In this situation, at an alpha level of 5 X 10^−08^, the CaNVAS sample of >11,400 stroke cases should provide 80% power to detect CNVs with ORs ranging from 1.14 to 1.32 across CNV frequencies ranging from 5% to 50%. Although stratification analyses are also planned, these by stroke subtype, ethnicity, sex, etc., and despite the heterogeneity of the samples, the given sample size will provide ample power.

### Functional annotation

Once outcome associated CNVs have been identified, functional annotational merging will be performed. The identified CNV loci will be mergered with the few other known stroke outcome loci including *BDNF*, *GPIIIa*, *COX2* [65] and the recently identified *PATJ* [66]. Overall, the functional annotation methods employed in these stroke outcome analyses will be consistent with those as described in Goal 1.

### Gene-based risk then pathway-based risk analyses

At this point in the analyses, single-CNV and gene-burden CNV results will be available. Again, methods consistent with the Goal 1 risk analyses will be employed, to identify pathways associated with stroke outcome. Based upon prior findings, ohnolog burden in specific pathways will be assessed.

At the completion of these stroke outcome-related analyses a list of ‘top’ CNVs, genes and pathways will be generated that will undergo biomarker evaluations consistent with those in Goal 2 using existing GeneStroke Consortium biomarker datasets. Available datasets include: proteomic data (52 cases and matching controls); epigenetic data (230 cases and matching controls); EWAS (n = 1072); miRNA(n = 260); RNA (n = 127; 40 of samples at 3 timepoints: 6 h, 24h and 3 months post stroke). Of note, these data are available on the same individuals used to identify the outcome-associated CNV, hence these analyses allow direct intra-subject correlation between CNV and the biomarker measures. While determining CNV-mediated relationships between case outcome and biomarker is the goal, control subjects biomarker levels can be used as baseline measures, as some biomarkers will likely change post-stroke.

### Potential problems and alternative strategies

The possibility exists that the efforts of Goal 1–3 may detect associations to CNV in genes whose products are not in the currently available in the TOPMed or GeneStroke biomarker datasets. Indeed, this was the case with the relatively recent discovery of an association between a variant in *HAPB2* and early-onset stroke [63]. In this case, other datasets were sought out in which it was demonstrated that FSAP, the protein product of *HAPB2*, correlated with the stroke risk allele yielding elevated FSAP levels [67]. Similarly, if CaNVAS detects such a CNV association, the possibility of developing a new assay for the product of this gene will be explored. Notably, biobanked blood is available from the majority of studies in CaNVAS, including GEOS, Krakow, Leuven, CADISP, SWEDEN, SIREN, and SLESS for such measurements in the event a new assay requires development.

### Junior investigator training network

Given the international structure of the grant and a desire to promote consistent collborative scientific involvement across all sites, a training network within the grant structure for junior researchers including Ph.D. Students and Post-Doctoral Research Fellows has been developed. Beyond providing detailed CNV methodological training, trainees will participate in all phases of the study, including the regularly-scheduled study conference calls. In addition to these monthly conference calls, periodic “journal clubs” will also occur, with senior investigators providing study-related lectures and seminars. Further, all trainees will attend at least one CaNVAS study meeting annually with these meetings timed to precede ISGC Workshops where they will present CaNVAS findings. Notably, the CNV techniques learned by the junior investigators can be applied to other future studies worldwide.

### Ethics statement

The authors declare that all relevant ethical guidelines have been followed, that all necessary IRB and/or ethics committee approvals have been obtained, that all necessary patient/participant consent has been obtained and that the appropriate institutional forms are archived. For all study subjects, the authors ensure that consent was informed and obtained via written consent. This study includes only adults, there are no minors. The authors confirm that this specific study was reviewed and approved before the study began by the Institutional Review Board of the University of Maryland, Baltimore (UMAB) through the UMAB Human Research Protections Office (HRPO)–email: hrpo@umaryland.edu (620 W. Lexington St., Second Floor, Baltimore, MD 21201 –Phone: 410-706-5037). UMAB HRPO Study ID#: HP-00087565. UMAB IRB review classified the study as: Type of IRB Review: Exempt; with a Determination Date: 8/16/2019.

### Timeline

**Fig 1:**
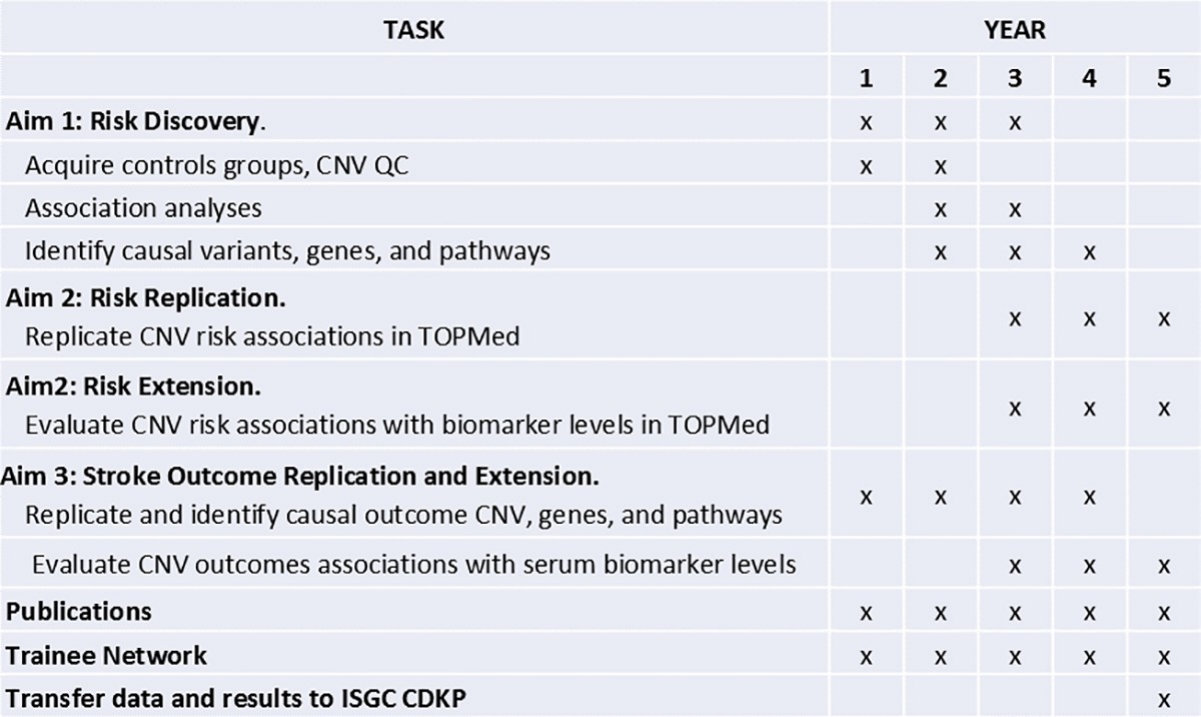
CaNVAS study timeline (2020–2025).

**Fig 1** demonstrates the CaNVAS timeline over the 5 year duration of the project.

### Initial data evaluation pilot study

Post-funding, an initial assessment of the data quality was perfumed reviewing 50 samples from each participating center using the PennCNV software package [53]. A comparison of the number of CNV-findings between the Centers was performed, as well as an analyses screening for outlier-cases with excessive number of CNV-calls. This is the only analyses presented in this manuscript.

## Results

### Initial data evaluation pilot study

**Fig 2:**
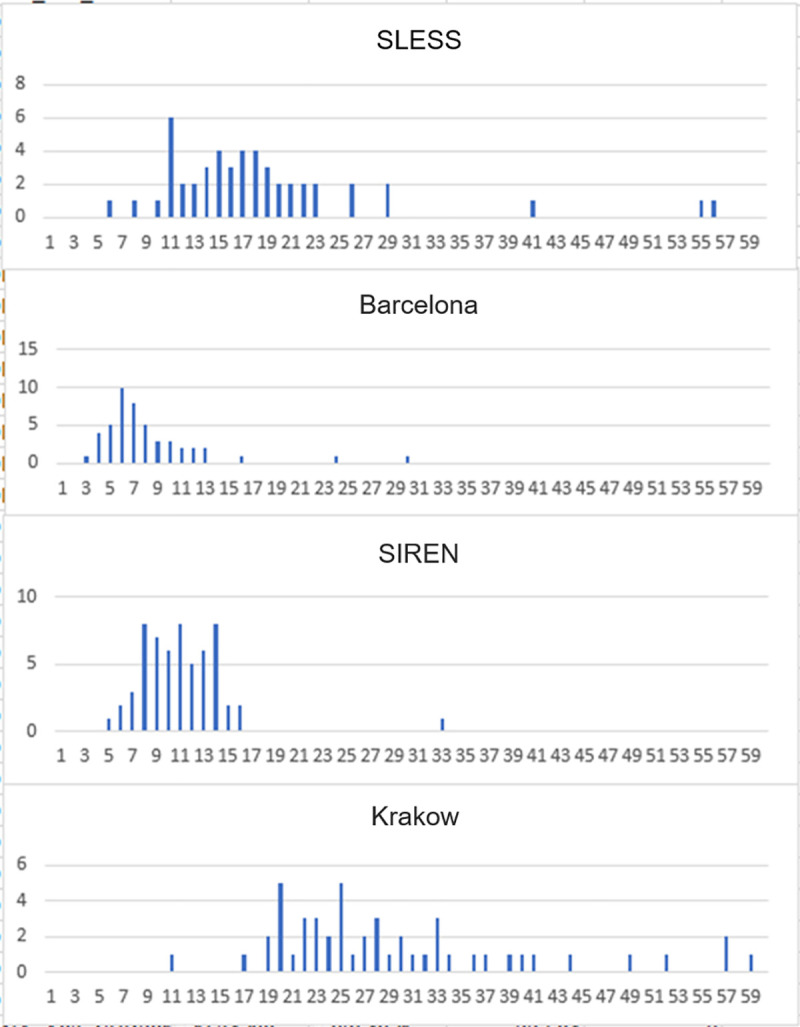
Histogram showing the number of CNV-calls by PennCNV (X-axis) and the number of samples (Y-axis) from 4 CaNVAS centers.

As described, CaNVAS uses existing GWAS and exome chip microarray samples from different platforms and different genotyping centers. To assess the quality of the data and to refine the analyses plan based on the differing data sets, a preliminary review of 50 samples from each participating center and microarray was performed. As shown in **Fig 2**, differing numbers of CNV were identified by the PennCNV software package [53] evaluating the 50 samples from each Center (e.g. 4 Centers shown in **Fig 2**). Such differences are not unexpected given the differing genotyping chips used as shown in **Table 2**.

As seen in **Fig 2**, the number of CNV-findings differs between the Centers (highest in Krakow and lowest in Barcelona). Moreover, this demonstrates that the number of CNV findings is similar for most cases from each Center, but that some outlier-cases exist with excessive number of CNV-calls.

The differences in the number of CNV calls is related to the SNP-density on the microarray. Krakow cases were analyzed with Illumina Exome Omni 5M chips, a high-density microarray with about 5,000,000 SNPs. The chips used for Barcelona had fewer SNPs, and therefore many smaller CNVs could not be detected, because for reliable detection of a CNV at least 5 consecutive SNPs should display increased (duplication) or decreased (deletion) signal intensities. The differences may also be related to the genomic location of the SNPs evaluated on a specific microarray, because CNV occurs preferably at particular genomic sites that may or may not have been included on any particular array.

**Fig 3:**
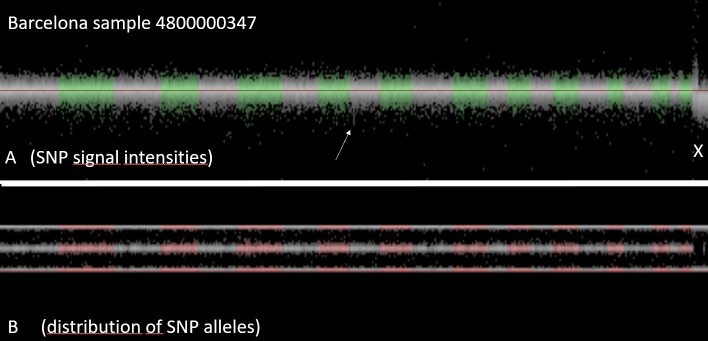
Visualization of all SNPs of a sample. Upper panel shows for each SNP the signal intensity, lower panel shows for each SNP the distribution of the signal across the two alleles. This case from Barcelona (Identifier 4800000347) is a man: the signal intensity of X-chromosomal SNPs is reduced (i.e. there is only one copy of the X-chromosome, compared to two copies of the autosomes. As there is only one X, there are no heterozygous SNPs. As a consequence, the mid-line of the allelic distribution (representing the heterozygous SNPs) is empty.

The variation of the number of CNV-calls within a center may be caused by technical error (purity of DNA, conditions of chip-hybridization and washing, etc.), but may also be related to biological causes, such as inbreeding or mosaicism. As a consequence, exclusion of all samples with outlier number of CNV is not recommended, since inbreeding or mosaicism may affect stroke risk and/or stroke outcome. Visual inspection of the samples revealed that the data quality was excellent for CNV analysis. The noise level was acceptable and most CNV were reliably detected. As example a case from Barcelona is illustrated in **Fig 3**.

**Fig 4:**
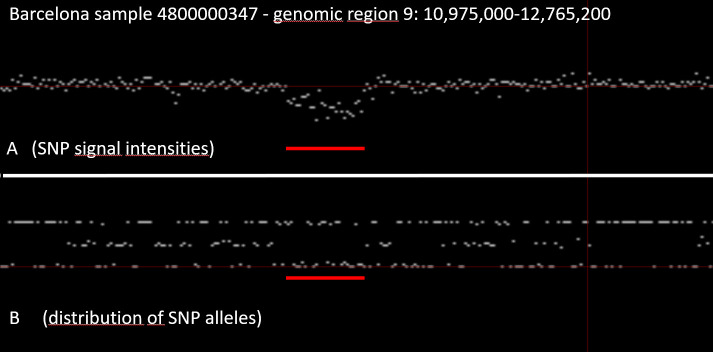
Detail of Fig 3 (arrow) region demonstrating a deletion in one of the two chromosomes.

**Fig 4** shows a detail of **Fig 3** (arrow), a zoomed in detail of chromosome 9. It is the region around the arrow in **Fig 3**. In this region, there is cluster of SNPs with characteristics like the X-chromosome (marked by the red bar in **Fig 4**). The signal intensity of all SNPs in this region is reduced, and none of the SNPs in this region are heterozygous. This finding suggests that there is only a single copy of this genomic region present in Barcelona case (Identifier 4800000437), i.e. this region is deleted in one of the two chromosomes. This interpretation is strengthened by the fact that a public database of human structural variation (DGV) reported a common deletion (esv3619645) of similar size in this region [68].

The identification of such a deletion is more difficult if the data is noisy. Moreover, platforms with a higher density of SNPs will have more SNPs within a CNV, which reduces the likelihood of false-positive findings. As such, the identification of larger CNVs, is more reliable compared to small CNVs. In genomic regions that are well represented on a microarray (high SNP-density) the detection of CNV is improved. As a consequence, many platforms were developed with additional SNPs in regions that are prone to CNV.

**Fig 5:**
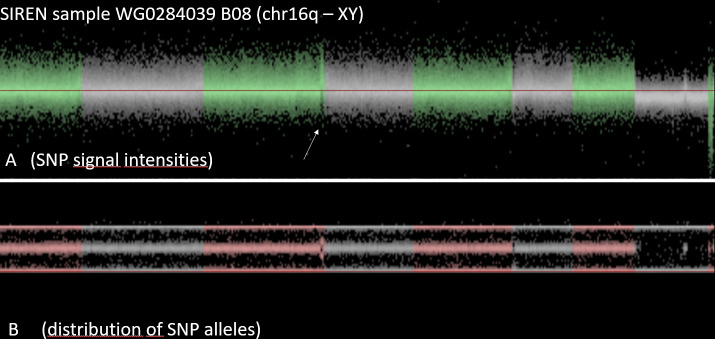
SIREN case with an additional copy of the genomic region at the tip of the long arm of chromosome 18 (duplication).

**Fig 6:**
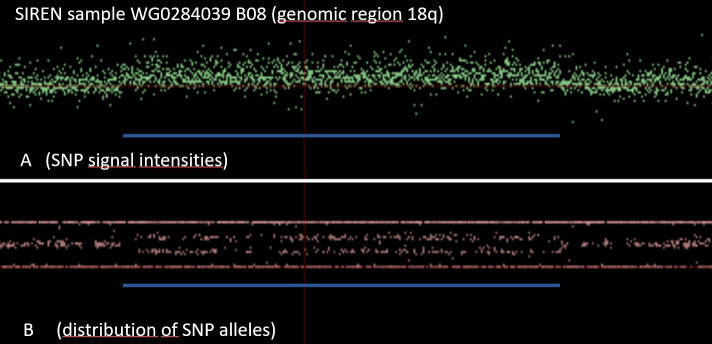
Zoom-in of the SIREN case with a CNV duplication in 18q.

As another example, **Fig 5** and **Fig 6** demonstrate a SIREN case with an additional copy of the genomic region at the tip of the long arm of chromosome 18 (duplication).

**Fig 7:**
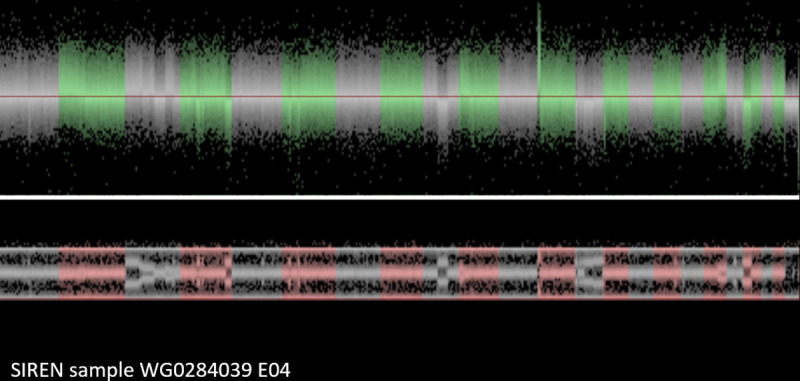
SIREN participant sample with clonal mosaicism.

Lastly, in **Fig 7**, another participant from the SIREN cohort demonstrates an outlier number of CNV calls as consistent with clonal mosaicism. Evaluating the visualization, this is simply not just a noisy case due to DNA degradation or some other technical error. Instead, the genome of this sample is enriched for many large chromosome aberrations, including duplications and deletions. Most of these were found in only part of the cells. Apparently, the blood cells, used for DNA extraction of this individual were not all identical. There were different cell lineages in this blood sample, with some of the cells carrying many structural aberrations. Such cases are not suitable for CNV analyses.

## Discussion

Overall, our pilot evaluation analyses demonstrate that the data are excellent for our planned CNV analyses. Further, as demonstrated by the SIREN participant with clonal mosaicism (**Fig 7**), some samples will be unsuitable for CNV analysis and can easily be identified and removed from further analyses. While the primary goals of CaNVAS are to identify and mechanistically understand CNV associated with ischemic stroke risk and outcome, numerous other studies are possible using the data that will be generated. Further, with the junior training network that is being developed, it is hoped that these data can be used toward PhD theses, among other projects.

As just described, visual inspection of the samples (for validation of CNV-findings) can lead to additional unexpected observations. For example:

1. Some individuals will have long genomic regions without heterozygous SNPs. Such long regions of homozygosity (LRoH) are typically caused by consanguinity: both parents transmitted the identical chromosome segment that was inherited from a common ancestor.

2. Some of the CNV-findings that were identified by the CNV-detection software programs can appear to be irregular, due to a particular type of abnormal signal intensities and allele distributions of the involved SNPs. These irregular CNV calls may be caused by clonal mosaicism as demonstrated in **Fig 7**. The DNA in such samples appears to be extracted from a mixed population of white blood cells, some with normal genotype, others with a CNV. Cell lineages with sex chromosome aberrations can be particularly frequent.

Preliminary studies suggested that both LRoH and mosaicism were associated with less favorable functional outcome after ischemic stroke ([69]; personal communication between Prapiadou S and Grond-Ginsbach C). As such, in the large CaNVAS study population, potential subprojects evaluating the effects of 1) inbreeding, and 2) mosaicism, on stroke outcome and on stroke risk are possible and would be of great interest.

Since CNV genotyping is difficult, a further subproject may analyze factors that determine CNV quality of GWAS microarrays. Microarray data may be very noisy, and it is not well understood why some samples are quite noisy, whereas others are not. Moreover, different types of noise can occur, including “genomic waves”, a systemic type of noise related to the GC content of the DNA. The microarrays that were genotyped at the Genome Resource Center (GRC) of the University of Maryland may be used to associated CNV quality with many technical items, including quality and quantity of DNA, position on the sample plate, genotype call rate in a previous GWAS, occurrence of mosaicism, amplitude of genomic waves, among others.

CaNVAS will collect a basic set of clinical data for stroke cases including age, sex, ethnicity, standard stroke risk factors, stroke subtype, NIHSS on admission, modified Rankin Score after 3 months (some cases 6 months), and for controls similar data including age, sex, ethnicity and standard vascular risk factors. Stratified analyses regarding specific variables are possible. Individual centers may have additional information about their patients and control subjects, including co-morbidities, socio-economic state, family history of vascular diseases, complications during hospitalization, brain imaging, laboratory parameters, etc. These data may allow a deeper analysis of the impact of CNV on stroke risk or stroke outcome.

## Conclusion

CaNVAS will cost-effectively leverage the numerous advantages of using existing case-control data sets, exploring the relationships between CNV and IS and its subtypes, and outcome at 3 months, in both men and women, in those of African and European-Caucasian descent, this, across the entire adult-age spectrum. For the newly discovered risk and outcome CNVs identified by CaNVAS, multiple bioinformatics approaches will be employed to identify the causal genes and affected pathways, merging these CNV loci in with the other known stroke susceptibility loci. The successful identification of novel genes, pathways and drug targets has the potential to transform our understanding of the stroke pathophysiology leading to more effective prevention and outcome strategies.
