# Supplementary material for: The copy number variation and stroke (CaNVAS) risk and outcome study
Source: PLoS One. 2021 Apr 19;16(4):e0248791. doi: 10.1371/journal.pone.0248791 (PMC8055008; doi:10.1371/journal.pone.0248791)
Supplement: Peer review history

# Editor report: Decision Letter 0

**DOI:** 10.1371/journal.pone.0248791.r001

**Authors:** Nancy Beam

5 Mar 2021

Copy Number Variation and Stroke (CaNVAS) Risk and Outcome: Study Protocol

PONE-D-21-04931

Dear Dr. Cole,

We’re pleased to inform you that your manuscript has been judged scientifically suitable for publication and will be formally accepted for publication once it meets all outstanding technical requirements.

Within one week, you’ll receive an e-mail detailing the required amendments. When these have been addressed, you’ll receive a formal acceptance letter and your manuscript will be scheduled for publication.

An invoice for payment may follow shortly after the formal acceptance. Since this is a commissioned article that has been approved for a fee waiver, please ignore this, but do let me know so I can notify billing. 

If your institution or institutions have a press office, please notify them about your upcoming paper to help maximize its impact. If they’ll be preparing press materials, please inform our press team as soon as possible -- no later than 48 hours after receiving the formal acceptance. Your manuscript will remain under strict press embargo until 2 pm Eastern Time on the date of publication. For more information, please contact onepress@plos.org.

Kind regards,

Nancy Beam, PhD

Staff Editor

PLOS ONE

# Editor report: Acceptance letter

**DOI:** 10.1371/journal.pone.0248791.r002

**Authors:** Nancy Beam

24 Mar 2021

PONE-D-21-04931 

The Copy Number Variation and Stroke (CaNVAS) Risk and Outcome Study. 

Dear Dr. Cole:

I'm pleased to inform you that your manuscript has been deemed suitable for publication in PLOS ONE. Congratulations! Your manuscript is now with our production department. 

If your institution or institutions have a press office, please let them know about your upcoming paper now to help maximize its impact. If they'll be preparing press materials, please inform our press team within the next 48 hours. Your manuscript will remain under strict press embargo until 2 pm Eastern Time on the date of publication. For more information please contact onepress@plos.org.

If we can help with anything else, please email us at plosone@plos.org. 

Thank you for submitting your work to PLOS ONE and supporting open access. 

Kind regards, 

PLOS ONE Editorial Office Staff

on behalf of

Dr. Nancy Beam 

Staff Editor

PLOS ONE
